# Price discovery in Chinese PVC futures and spot markets: Impacts of COVID-19 and benchmark analysis

**DOI:** 10.1016/j.heliyon.2024.e24138

**Published:** 2024-01-11

**Authors:** Yunyan Wu, Jiaqi He, Linfei Xiong

**Affiliations:** a School of Business, Jianghan University, Shatin, Wuhan, PR China; b Department of Financial Statistics, Zhongnan University of Economics and Law, Wuhan, PR China

**Keywords:** Price discovery, PVC futures market, PVC spot market, Component share, Information share, Information leadership share

## Abstract

Despite being a minor futures category, Polyvinyl chloride (PVC) futures have emerging as a vital element in energy and chemical futures domain. Employing three benchmark models component share (CS), information share (IS), and information leadership share (ILS), this study explores the price discovery function of Chinese PVC futures and spot markets. It assesses whether PVC futures have matured into an effective hedging tool and reference point for spot markets, and also examines the impact of the COVID-19 pandemic on this price discovery relationship. Empirical analysis reveals that the futures market has become the primary site for price discovery in the Chinese PVC market. All the models consistently demonstrate a mature price discovery function in PVC futures, providing risk mitigation tools for industry players. However, post-pandemic dynamics indicate that price discovery in PVC markets primarily occurs within the spot market. This suggests that compared to the futures market, the PVC spot market is able to respond more quickly to the strong signals of industrial recovery after the end of the pandemic. The feedback and pricing efficiency of the PVC futures market in response to new market information are also influenced. Furthermore, our study offers better anticipation of future market prices.

## 1 Introduction

The energy and chemical industry, epitomized by petroleum and coal, stands as a pivotal sector in China. Over the past decade, the futures market within this domain has experienced rapid expansion, witnessing a continuous influx of listed commodities. In 2022, the trading volume of China's energy and chemical futures market reached an impressive 15.87 trillion yuan, constituting 30.7% of the total annual trading volume. Presently, energy and chemical futures have evolved into the third-largest category of futures, trailing behind agricultural products and metallic commodities. PVC, a vital synthetic resin, boasts commendable physical and chemical attributes, finding extensive utility across industrial, packaging, and daily-life. Globally, PVC consumption falls second only to polyethylene among the major general-purpose resins. Developed nations, drawing on their abundant petroleum, shale gas, and natural gas resources, predominantly employ the ethylene-based process for PVC production. This method ensures uniform and pure product quality, predominantly targeting middle and high-end markets. In contrast, due to China's resource composition characterized by “rich coal, scarce oil, and limited gas,” the prevalent method for PVC production relies on the calcium carbide route, catering to the middle and lower-end markets. In 2021, China's PVC exports totaled 1.75 million tons, accounting for approximately 21.9% of the global export volumes [1]. At present, only China and a handful of other nations primarily adopt the calcium carbide route for PVC production, resulting in discernible disparities in production costs and market prices compared to their counterparts in Europe and the United States. Simultaneously, PVC futures, classified as a minor variety within the futures market, exhibit significantly lower trading volumes and turnover when juxtaposed with bulk commodities such as crude oil, gold, and silver. In 2022, the total annual trading volume of China's PVC futures market amounted to 28.366 million contracts, positioning it as the sixth largest within the energy and chemical futures domain. In light of global energy structural adjustments, shifts in the supply-demand landscape of the petrochemical market, trade protection policies, and the repercussions of the COVID-19 pandemic, the PVC futures and spot markets confront substantial opportunities and challenges. The unexpected start-stop cycles prompted by these macroscopic events can engender profound economic losses and environmental safety concerns for PVC manufacturers, while the tumultuous fluctuations in market prices pose considerable risks for participants in PVC-related financial markets. Thus, the interconnectivity between PVC futures and spot markets bears considerable pragmatic significance for manufacturers, investors, and researchers alike.

Price discovery is fundamental to the development of futures markets and the practice of hedging transactions [2], [3]. It reflects the ability of futures markets to swiftly adjust prices upon the influx of new information and promptly respond to market changes. An efficiently operating futures market possesses a mature price discovery function, offering reference prices for spot markets and influencing supply-demand dynamics and price formation in spot markets through hedging transactions and other means [4], [5]. With advantages such as high leverage, high liquidity, low transaction costs, and short selling mechanisms, futures markets, in general consensus among scholars, tend to exhibit more sensitive responses to information compared to spot markets [6], [7]. They have the capacity to steer price changes in spot markets, enabling investors to mitigate risks through hedging transactions and thereby reducing portfolio risks. The process of price discovery between futures and spot markets is dynamic [8], [9]. During this process, fluctuations in spot market prices and supply-demand relationships can directly or indirectly impact the futures market [10], [11]. Conversely, price fluctuations in the futures market and participants' trading behaviors can also influence the spot market. Through the interaction between futures and spot markets, market participants can capitalize on information and price differentials for arbitrage and risk management, ultimately fostering equilibrium prices between the two markets [12]. Nevertheless, there remains a dearth of research both domestically and internationally, especially pertaining to the context of macroscopic events, regarding price discovery in PVC futures and spot markets. This scarcity underscores the significance of investigating price discovery within China's PVC market, rendering it a highly meaningful research subject.

Previous scholars' theoretical research has primarily focused on analyzing the price discovery mechanisms of commodities with significant trading volumes within specific time frames [13], [14], [15]. Jin et al. (2018) employed the CS model, IS model, and ILS model to examine the price discovery between Chinese gold markets. They concluded that the results of the IS model and ILS model align with the CS model, highlighting the dominant role of the gold futures market in the price discovery process [16]. Hao et al. (2021) investigated the price discovery of the Chinese soybean option market and futures market using the CS model and IS model. Their findings indicated that the contribution to price discovery from the soybean option market surpasses that of the futures market [17]. Shao and Hua (2022) utilized the CS model, IS model, and a modified version of the IS model to study price discovery in the West Texas Intermediate, Brent, and Chinese crude oil markets. Discrepancies arose between the research outcomes of the IS model and CS model, with the modified IS model showing similar conclusions to the IS model. However, the modified IS model failed to reconcile the contradictory results between the IS and CS models [18]. For minor futures markets such as the PVC futures market, there has been relatively limited research into its market characteristics and price discovery function [19]. Due to restrictions imposed by coal, lake salt, and well salt resources, over 80% of China's PVC production capacity employs the calcium carbide process, which significantly differs from the ethylene-based process predominantly used in Western countries [20]. This divergence results in unique market characteristics and price differentials within the Chinese PVC market. Simultaneously, upstream PVC producers generally comprise large state-owned enterprises or well-established private enterprises. They operate on a larger scale with fewer entities, engage in continuous production, possess some pricing power, but experience significant production and cost pressures. Conversely, downstream users of PVC products usually consist of smaller enterprises operating in larger numbers, conducting intermittent production, lacking pricing power, but facing lesser production pressures. These features lead to frequent substantial disagreements between supply and demand parties regarding prices, contributing to complex price fluctuations in both the PVC futures and spot markets.

Since the introduction of PVC futures in China, research into the price discovery function between PVC markets has been notably limited. The outbreak of the COVID-19 pandemic in December 2019, its subsequent spread within China and globally, disruptions in industry supply chains, decreased consumer demand, market price volatility, and changes in import-export policies have collectively exerted undeniable impacts on the PVC industry. However, with the gradual vaccination efforts and the global economic recovery, China declared the end of the COVID-19 pandemic in December 2022. This declaration holds the promise of a gradual recovery of industrial production, including the PVC industry, and its adaptation to new market conditions. Recently, many studies are exploring the impact of the significant event of the COVID-19 pandemic on financial markets, particularly in the futures market. Wu et al. [21] employed the fractional cointegrated vector autoregressive model to assess the high-frequency price discovery in bitcoin markets, revealing a shift in leadership from the futures market to the spot market during the Covid-19 pandemic. Shrestha et al. [22] investigated the impact of the COVID-19 pandemic on the price discovery of four carbon exchange-traded funds markets, finding that the iShares MSCI ACWI Low Carbon Target (iShare) ETF dominated the pre-COVID price discovery, but its contribution substantially declined during the pandemic. Yu et al. [14] explored the time-varying price discovery patterns of China's INE crude oil futures during the COVID-19 pandemic shock, noting a significant impact on the process, with a slight improvement in performance post-recovery. Mohamad et al. [23] assessed the price discovery contribution of bitcoin spot and futures markets using information share metrics, revealing a shift in leadership from bitcoin futures to the spot market during the pandemic. Luu et al. [24] analyzed daily short-selling activities in the U.S. market during the early 2020 outbreak of the global COVID-19 pandemic, highlighting the significant role of short selling in enhancing price discovery, supporting its substantial contribution during the pandemic. Consequently, a thorough investigation is warranted to ascertain whether the Chinese PVC futures market possesses a mature price discovery function and whether the PVC futures market has been able to operate efficiently, stabilize market prices, and provide reliable reference prices pre and post the COVID-19 pandemic.

It is also noted that research on the price discovery of futures and spot markets is closely connected to many key financial theories such as market microstructure theory [25] and efficient market hypothesis (EMH) [26]. Market microstructure theory focuses on the process of information transmission within the market, as well as the transparency and efficiency of the market. A highly efficient market structure contributes to more effective price discovery, as market participants can interpret and respond to information more accurately. Research on the price discovery of futures and spot markets typically considers the transmission of information between these markets and the impact of market structure on information flow, aligning closely with market microstructure theory [27]. EMH posits that markets reflect all available information, making it challenging to achieve long-term excess returns beyond the market average [28]. This theory provides a conceptual foundation for understanding market efficiency and information reflection. Research on the price discovery of futures and spot markets serves to empirically validate the applicability of this theoretical framework across different markets. Market microstructure theory and EMH offer financial theory foundations for comprehending the price discovery of futures and spot markets.

This study examines the price discovery function between PVC futures and spot markets in China since the introduction of PVC futures. It also conducts a dynamic analysis of the price discovery relationship between PVC futures and spot markets before and after the outbreak of the COVID-19 pandemic. The research findings indicate that following the introduction of PVC futures, the primary occurrence of price discovery in the Chinese PVC market takes place within the futures market. The results from the CS, IS, and ILS models are consistent, indicating an overall mature price discovery function in the PVC futures market. This function serves as a hedging tool for the real economy, enabling producers and consumers to proactively lock in future prices. Enterprises can safeguard against market volatility risks by trading futures contracts to protect the prices of raw materials or products. The COVID-19 pandemic has impacted both the upstream and downstream segments of China's PVC industry to varying degrees. This study calculates the price discovery relationships in the PVC futures and spot markets before, during, and after the pandemic. The aim is to further examine the price discovery function of China's PVC futures market. The results reveal that, for the nationwide ethylene-based spot market, nationwide calcium carbide spot market, and the eastern region's calcium carbide spot market, following the end of the pandemic, price discovery in the PVC market primarily occurs in the spot market. This suggests that post-pandemic, the PVC futures market has not yet achieved a highly efficient operational state, unable to swiftly respond to the impact of new market information and changes. In the price discovery process, the PVC spot market assumes a dominant role. Furthermore, due to the strong correlation in residual sequences between PVC futures and spot markets, after eliminating the correlation, the modified IS model effectively resolves the contradictory price discovery outcomes between the CS and IS models. This study contributes to a deeper understanding of the operational mechanism and price formation process in the PVC futures market. It offers valuable insights for market participants to better anticipate future product market prices. Additionally, it provides theoretical evidence for the price guidance relationship between PVC futures and spot markets, further enriching the theoretical research within the PVC derivatives market.

The rest of the paper is organized as follows. Section 2 presents the measures of price discovery and detail the methods used in the paper. Section 3 is devoted to the description and summary statistics of the data used in our study. Section 4 presents the empirical results and our main findings. The conclusion of the paper is given in Section 5.

## 2 Methodology

Three models are adopted to calculate the price discovery relationship between PVC futures and spot markets. The Component Share (CS) model and Information Share (IS) model [29] are based on the Vector Error Correction Model (VECM) and calculate the price discovery relationship between the futures and spot markets based on different definitions of price discovery. The CS model is based on the market's error correction coefficients, suggesting that in the error correction process, the contribution of each market to the common factors represents the proportion of price discovery in the futures and spot markets. The CS model only considers permanent asset price shocks in the price discovery process. The IS model defines price discovery as the variance of new information on common factors. IS measures the relative contribution of futures and spot markets to the variance, representing the proportion of price changes contributed by the futures and spot markets [30], [31]. When the residual sequences between the two markets are unrelated, the IS and CS models provide similar conclusions regarding price discovery. However, when there is correlation between the residual sequences of the two markets, it can lead to paradoxical results for the IS and CS models [32], [33]. Therefore, when using the IS model to calculate the price discovery relationship between futures and spot markets, it is necessary to consider excluding the correlation between the residual sequences and make adjustments to the IS indicator.

It is believed that noise in the market and the speed of market reactions to new information are the main factors affecting the price discovery ability of futures and spot markets. Only when the price sequences of the two markets have similar noise levels, can the IS and CS models accurately measure the price discovery between the markets. To address this issue, Information Leadership Share (ILS) model is for calculating the market's price discovery ability [34], [35]. The ILS model combines the results of the IS and CS models and effectively eliminates the impact of noise on price discovery.

### 2.1 Component share

The VECM (Vector Error Correction Model) is a time series method used to study the long-term equilibrium relationship and short-term dynamic adjustment process among multiple sequences. The model introduces error correction terms to consider the long-term equilibrium relationship between the sequences. If there exists a long-term equilibrium relationship among multiple sequences, the error correction mechanism between the sequences will gradually bring the system back to an equilibrium state. When conducting price discovery research between futures and spot markets, if both futures and spot prices are first-order cointegrated sequences, constructing a VECM model allows for the calculation of the contribution of price discovery between different markets. The general form of the VECM model is as follows:

(1) $$△X_{t}=\alpha \beta^{\prime }X_{t-1}+\sum_{i=1}^{k}A_{i}△X_{t-i}+\epsilon_{t},$$

where $X_{t}={\left(x_{1,t},x_{2,t}\right)}^{\prime }$, $x_{1,t}$ and $x_{2,t}$ denote the logarithms of the future price and spot price. $△X_{t-i}$s (i=0,1,…,k) represent the log returns of the futures and spot markets. *α* is the error-correction vector used to measure the speed of adjustment. *β* is the cointegrating vector ${\left(1,-1\right)}^{\prime }$. $A_{i}$s are the common factor coefficient vectors. $\epsilon_{t}$ is the zero-mean vector of innovation with following correlation matrix

(2) $$\Omega =\left(\begin{array}{cc} \sigma_{1}^{2}\; & \rho \sigma_{1}\sigma_{2}\\ \rho \sigma_{1}\sigma_{2}\; & \sigma_{2}^{2} \end{array}\right),$$

where $\sigma_{1}^{2}$ is the variance of PVC futures, $\sigma_{2}^{2}$ is the variance of PVC spot, $\rho \sigma_{1}\sigma_{2}$ is the covariance between PVC futures and spot.

In the CS model, if there is a price discovery relationship between two markets, the differences in the transmission efficiency of new information between the markets can cause price imbalances between the futures and spot markets [36]. The common factor represents the component where common and efficient information is permanently incorporated into prices in both futures and spot markets. To measure the contribution of both markets to the common factor, the CS model uses the orthogonal vector *γ* ($\gamma =\alpha_{⊥}={\left(\gamma_{1},\gamma_{2}\right)}^{\prime }$) of the error correction coefficient vector for calculation. In VECM, the orthogonal vector of the error correction coefficient vector measures the common and efficient prices of the futures and spot markets during the price discovery process. Let

(3) $$CS_{1}=\frac{\left|\gamma_{1}\right|}{\left|\gamma_{1}|+|\gamma_{2}\right|},\;\;\;\;\;\;\;\;CS_{2}=\frac{\left|\gamma_{2}\right|}{\left|\gamma_{1}|+|\gamma_{2}\right|},$$

where $\gamma_{1}=\frac{\alpha_{2}}{\alpha_{2}-\alpha_{1}}$ and $\gamma_{2}=\frac{\alpha_{1}}{\alpha_{1}-\alpha_{2}}$. $CS_{1}$ and $CS_{2}$ depict the contribution of the futures and spot markets to the common factor during the price discovery process, representing the permanent asset price shocks in the process. If the futures market completely dominates the price discovery in the spot market, $CS_{1}=1$; when the spot market plays an absolute dominant role in price discovery, $CS_{2}=1$. A higher component share value indicates a greater contribution of the corresponding market to the price discovery process.

### 2.2 Information share

Price discovery is defined as that the contribution of the innovations on futures and spot markets to the total variance [37]. In the IS model, the total variance of innovation is decomposed, and when there is no correlation between the variances of innovations, the calculation of the information share of futures and spot markets is as follows:

(4) $$IS_{1}=\frac{\gamma_{1}^{2}\sigma_{1}^{2}}{\gamma_{1}^{2}\sigma_{1}^{2}+\gamma_{2}^{2}\sigma_{2}^{2}},\;\;\;\;\;\;\;\;\;IS_{2}=\frac{\gamma_{2}^{2}\sigma_{2}^{2}}{\gamma_{1}^{2}\sigma_{1}^{2}+\gamma_{2}^{2}\sigma_{2}^{2}}.$$

When there is a correlation between the variances of innovations, to eliminate contemporaneous correlations between the sequences, the covariance matrix Ω is typically subjected to Cholesky factorization. In this case, the calculation of price discovery between futures and spot markets is as follows:

(5) $$IS_{1}=\frac{{\left(\gamma_{1}n_{11}+\gamma_{2}n_{12}\right)}^{2}}{{\left(\gamma_{1}n_{11}+\gamma_{2}n_{12}\right)}^{2}+{\left(\gamma_{2}n_{22}\right)}^{2}},\;\;\;\;\;IS_{2}=\frac{{\left(\gamma_{2}n_{22}\right)}^{2}}{{\left(\gamma_{1}n_{11}+\gamma_{2}n_{12}\right)}^{2}+{\left(\gamma_{2}n_{22}\right)}^{2}},$$

where

(6) $$N=\left(\begin{array}{cc} n_{11}\; & 0\\ n_{12}\; & n_{22} \end{array}\right)=\left(\begin{array}{cc} \sigma_{1}\; & 0\\ \rho \sigma_{2}\; & \sigma_{2}{\left(1-\rho^{2}\right)}^{1/2} \end{array}\right).$$

Due to Cholesky factorization potentially overemphasizing the information share of the market listed first, when using the IS model to calculate the price discovery relationship between futures and spot markets, one can modify the market order in Cholesky factorization. By calculating the upper and lower bounds of the information share and taking the average value, the final result represents the measure of price discovery functionality between the markets.

Bailliea (2002) research found that when the residual sequences between two markets are unrelated, the IS and CS models provide similar conclusions regarding price discovery. However, when there is a clear correlation between the residual sequences of the two markets, it can lead to paradoxical results for the IS and CS models. Therefore, to avoid the impact of residual sequence correlation on market information share, when using the IS model to calculate the price discovery relationship between futures and spot markets, it is necessary to first eliminate the correlation between the residual sequences and make adjustments to the IS indicator. Using the least squares method [38], we remove the correlation between the residual sequences of futures and spot markets. After eliminating the correlation, the residuals corresponding to futures and spot markets are denoted as $\epsilon_{f,t}$ and $\epsilon_{s,t}$, respectively.

(7) $$(\epsilon_{f},\epsilon_{s})=(\epsilon_{1t},\epsilon_{2t}-\rho \frac{\sigma_{2}}{\sigma_{1}}\epsilon_{1t}).$$

After removing the correlation, the IS model is used again to calculate the contribution of futures and spot markets to the variance of the common factor innovation. This approach resolves the paradoxical results between the CS model and the IS model, allowing for an effective measurement of the price discovery relationship between the two markets. The IS model decomposes the variance of the common factor, quantifying each market's contribution to the variance of the common factor when facing new information shocks. It measures the short-term deviations of futures and spot prices.

### 2.3 Information leadership share

Based on the research by Yan and Zivot (2010), Putnins (2013) proposed the ILS (Information Leadership Share) model to calculate the price discovery relationship between markets. Putnins argued that only when the price sequences have equal levels of noise (including microstructure frictions and market liquidity), the CS and IS models can effectively depict the market's price discovery ability. The calculation method of ILS is as follows:

(8) $$ILS_{1}=\frac{\frac{IS_{1}}{IS_{2}}\cdot \frac{CS_{2}}{CS_{1}}}{\frac{IS_{1}}{IS_{2}}\cdot \frac{CS_{2}}{CS_{1}}+\frac{IS_{2}}{IS_{1}}\cdot \frac{CS_{1}}{CS_{2}}},\;\;\;\;\;\;ILS_{2}=\frac{\frac{IS_{2}}{IS_{1}}\cdot \frac{CS_{1}}{CS_{2}}}{\frac{IS_{1}}{IS_{2}}\cdot \frac{CS_{2}}{CS_{1}}+\frac{IS_{2}}{IS_{1}}\cdot \frac{CS_{1}}{CS_{2}}}.$$

The ILS model combines the component share and information share to determine the price sequence that absorbs market new information first. Putnins pointed out that the CS and IS models can provide misleading information about price discovery under certain circumstances. By integrating the CS and IS models, the ILS model effectively eliminates the dependency on noise in both indicators, causing the dynamic responses of price sequences to transitory shocks to cancel out, while retaining the dynamic response proportion of price sequences to permanent shocks. When $ILS_{i}>0.5$, the corresponding market plays a dominant role in the price discovery process.

## 3 Data description

### 3.1 Data and descriptive statistics

**Figure 1:**
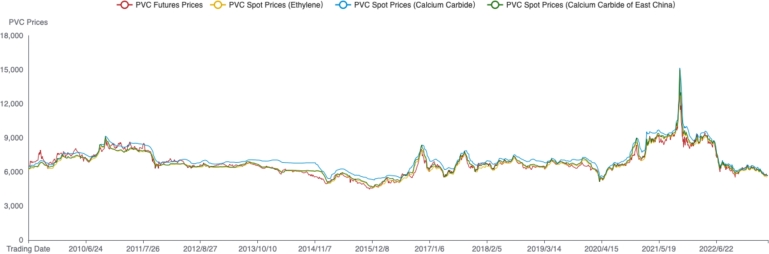
Futures and spot prices of China's PVC markets. This gure displays the daily prices of China's PVC futures and spot markets. The red, orange, blue and green lines represent the futures prices, the spot prices of PVC ethylene markets, the spot prices of PVC calcium carbide markets and the spot prices of PVC calcium carbide markets in East China, respectively. PVC futures prices and spot prices have similar price trends.

China's PVC futures were listed on May 25, 2009, on the Dalian Commodity Exchange. This study selects data from PVC futures and spot markets since the listing date as the research objects for PVC market price discovery. The futures data consists of the closing prices of active PVC futures contracts from May 25, 2009, to June 13, 2023, totaling 3418 trading days. The spot data represents the average price of PVC in the East China region, produced using the calcium carbide and ethylene methods. The East China region is a major consumption area for PVC in China, making it a suitable choice for analyzing spot prices. The data is sourced from the Wind database. The daily price changes of PVC futures and spot prices are shown in Fig. 1. Overall, the price trends and variations in PVC futures and spot prices are quite similar.

The descriptive statistical analysis results for PVC futures and spot prices are presented in Table 1. The coefficients of variation for PVC futures price, Chinese Ethylene Spot price, Chinese Calcium Carbide Spot price, and East China Calcium Carbide Spot price are 0.1598, 0.1570, 0.1612, and 0.1607, respectively. This indicates that, on the whole, compared to the futures market, the prices in the Chinese Calcium Carbide spot market and the East China Calcium Carbide spot market exhibit greater volatility, while the PVC futures market prices remain relatively stable. The correlation coefficients between PVC futures and spot prices are 0.9458, 0.9705, and 0.9704, indicating a strong correlation in price movements between the markets. This suggests the possibility of a long-term, stable equilibrium relationship between PVC futures and spot markets.

**Table 1:** Descriptive statistics of PVC futures and spot markets.

Variable	Min	Max	Mean	SD	CV	Skewness	Kurtosis
Futures	4445	13215	6770	1081.90	0.1598	0.8622	1.8711
Spot (Ethylene)	5283	15150	7162	1124.72	0.1570	1.6524	6.0594
Spot (Calcium carbide)	4632	14610	6736	1086.04	0.1612	1.3103	4.0139
Spot (Calcium carbide	4690	14975	6827	1096.73	0.1607	1.3849	4.3391
of East China)							

Note: The values represent the minimum, maximum, mean, standard deviation, coefficient of variation, skewness and kurtosis of PVC futures and spot prices. Spot prices are PVC prices in China's ethylene and calcium carbide markets, with a separate entry for calcium carbide prices in East China.

**Figure 2:**
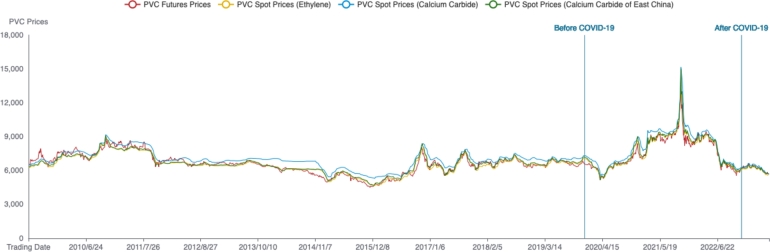
Futures and spot prices of PVC markets before and after COVID-19. The gure displays the trends of futures and spot prices in China's PVC markets, with a clear distinction before and after the COVID-19 pandemic.

In order to comprehensively study the price discovery relationship between PVC futures and spot markets, this paper further examines the price discovery relationship between the PVC markets before and after the epidemic. Based on the significant time points marking the beginning and end of the epidemic in China, the PVC data is divided. The period before the epidemic is from May 25, 2009, to December 11, 2019, totaling 2568 trading days; the epidemic period is from December 11, 2019, to December 4, 2022, totaling 724 trading days; the period after the epidemic is from December 5, 2022, to June 13, 2023, totaling 126 trading days. Fig. 2 presents a comparative graph of PVC futures and spot prices before and after the epidemic.

Before and after the epidemic, the descriptive statistical analysis results of PVC futures and spot prices are shown in Table 2. Before the epidemic, the coefficient of variation for PVC futures market prices was 0.1314, which was higher than the coefficient of variation for the PVC spot market. This indicates that before the epidemic, the PVC futures market exhibited higher price volatility, while the spot market's prices were relatively stable. During the epidemic period, the price volatility of PVC in all three spot markets was higher than that of the PVC futures market. After the epidemic, the price volatility of the ethylene-based spot market was the highest, while the volatility of prices in the calcium carbide-based spot market in the East China region was the lowest.

**Table 2:** Descriptive statistics of PVC markets before and after COVID-19.

Time division	Variable	Min	Max	Mean	SD	CV	Skewness	Kurtosis
Before	Futures	4445	9100	6564	862.34	0.1314	-0.0014	-0.0107
COVID-19	Spot (Ethylene)	5283	9162	6930	746.70	0.1078	0.1473	0.3982
	Spot (Calcium carbide)	4632	8878	6496	776.64	0.1196	0.0378	0.2825
	Spot (Calcium carbide	4690	14975	6827	1096.73	0.1607	0.0692	0.2786
	of East China)							

During	Futures	5040	13215	7608	1401.23	0.1842	0.4973	0.0053
COVID-19	Spot (Ethylene)	5475	15150	8058	1637.72	0.2011	0.8270	1.6339
	Spot (Calcium carbide)	5135	14610	7703	1484.33	0.1927	0.6836	0.9562
	Spot (Calcium carbide	5150	14975	7823	1510.71	0.1931	0.6952	1.0707
	of East China)							

After	Futures	5638	6696	6163	250.51	0.0406	-0.5447	-0.5256
COVID-19	Spot (Ethylene)	5675	6655	6244	272.69	0.0437	-0.7357	-0.5467
	Spot (Calcium carbide)	5532	6442	6061	241.04	0.0398	-0.9265	-0.2684
	Spot (Calcium carbide	5625	6540	6136	241.80	0.0394	-0.7152	-0.5678
	of East China)							

Note: The values represent the minimum, maximum, mean, standard deviation, coefficient of variation, skewness and kurtosis of PVC futures and spot prices. The table presents descriptive statistics of PVC market variables before, during and after the COVID-19 pandemic.

**Figure 3:**
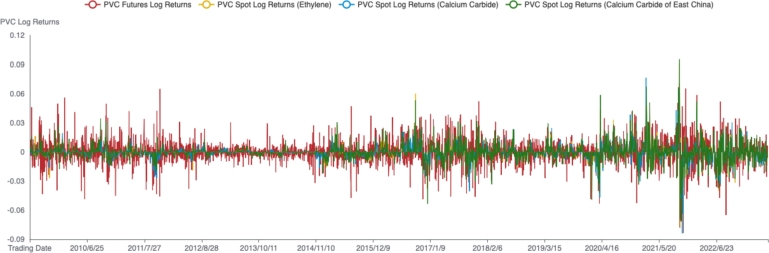
Futures and spot logarithmic return rate of PVC markets. The red, orange, blue and green lines represent the trends of logarithmic return rates of futures and spot prices in China's PVC markets, respectively.

The comparative graph of the logarithmic returns of PVC futures and spot markets, as well as the descriptive statistical analysis results, is presented in Fig. 3 and Table 3, respectively. The coefficient of variation of the logarithmic returns in the PVC futures market is much higher than that in the spot market, indicating that the returns in the PVC futures market exhibit more pronounced volatility compared to the spot market. The distribution of returns in the PVC futures market is left-skewed, suggesting a higher risk of declining returns in the futures market. On the other hand, the distribution of returns in the spot market is right-skewed, with the calcium carbide-based spot market showing a more pronounced right-skewness. This indicates that the calcium carbide-based spot market has more trading days where returns are higher than its own mean. Meanwhile, both PVC futures and spot markets' returns exhibit the characteristics of leptokurtosis (fat-tailedness). However, the kurtosis of returns in the PVC spot market is significantly higher than that in the PVC futures market, indicating that the microstructures of different PVC markets are distinct, and there might be variations in the speed at which different markets absorb and reflect new information.

**Table 3:** Descriptive statistics of PVC logarithmic return rate.

Variable	Min	Max	Mean	SD	CV	Skewness	Kurtosis
Futures	-0.0835	0.0709	-3.89e-05	0.0129	-332.5324	-0.1067	4.2249
Spot (Ethylene)	-0.0831	0.0767	-3.65e-05	0.0064	-174.5962	0.4262	38.5543
Spot (Calcium carbide)	-0.0772	0.0911	-3.43e-05	0.0073	-211.6342	0.6594	26.6742
Spot (Calcium carbide	-0.0712	0.0953	-3.27e-05	0.0082	-250.8769	0.6292	19.1896
of East China)							

Note: This table presents descriptive statistics of the logarithmic return rates for PVC futures and spot markets.

### 3.2 Augmented Dickey-Fuller stationary test

We conducted stationary tests on the original PVC futures and spot market prices, as well as the logarithmic returns of PVC futures and spot markets. The original PVC futures and spot price sequences are both non-stationary, while the logarithmic return sequences of the PVC markets are stationary. Therefore, the original futures and spot market sequences are both first-order integrated I(1) sequences. The results of the stationarity tests for the PVC markets are presented in Table 4.

**Table 4:** Stationarity test results of PVC markets.

Variable	All data	Before	During	After
		COVID-19	COVID-19	COVID-19
Futures prices	-0.5354	-0.2428	-0.3426	-0.5039
	(0.59)	(0.81)	(0.73)	(0.62)
Futures log returns	-39.6711	-34.5116	-17.8861	-8.8625
	(0.00)	(0.00)	(0.00)	(0.00)
Spot prices (Ethylene)	-0.4918	0.2503	-0.4507	-0.8712
	(0.62)	(0.80)	(0.65)	(0.39)
Spot log returns	-25.5596	-22.8867	-11.2562	-6.8899
(Ethylene)	(0.00)	(0.00)	(0.00)	(0.00)
Spot prices	-0.5272	0.2108	-0.4739	-0.9833
(Calcium carbide)	(0.60)	(0.83)	(0.64)	(0.33)
Spot log returns	-30.1862	-26.1714	-13.9123	-7.3728
(Calcium carbide)	(0.00)	(0.00)	(0.00)	(0.00)
Spot prices (Calcium	-0.5510	0.0844	-0.4856	-0.8156
carbide of East China)	(0.58)	(0.93)	(0.63)	(0.42)
Spot log returns (Calcium	-31.7078	-26.0471	-14.7476	-8.1661
carbide of East China)	(0.00)	(0.00)	(0.00)	(0.00)

Note: The table denotes the ADF test results of stationarity tests for PVC market variables. The values are reported as test statistics followed by p-values in parentheses. If p<0.05, it denotes that the corresponding price series is stationary.

### 3.3 Johansen cointegration test

We conducted cointegration tests on the PVC futures and spot price sequences using the Johansen test. The trace test and the maximum eigenvalue test both indicate the presence of 1 cointegration relationship between PVC futures and spot prices. Therefore, it is possible to conduct a price discovery study on PVC futures and spot markets by establishing a Vector Error Correction Model (VECM). The results of the cointegration test for the PVC markets are presented in Table 5.

**Table 5:** Trace test of PVC markets.

Time division	Variable	Hypothesis	Statistics	10%	5%	1%
				Critical	Critical	Critical
				value	value	value
All data	Spot (Ethylene)	*H*_0_:*r* = 0	46.07	17.85	19.96	24.60
		*H*_1_:*r* = 1	8.51	7.52	9.24	12.97
	Spot (Calcium carbide)	*H*_0_:*r* = 0	62.84	17.85	19.96	24.60
		*H*_1_:*r* = 1	8.07	7.52	9.24	12.97
	Spot (Calcium carbide	*H*_0_:*r* = 0	51.23	17.85	19.96	24.60
	of East China)	*H*_1_:*r* = 1	6.77	7.52	9.24	12.97

Before	Spot (Ethylene)	*H*_0_:*r* = 0	41.78	17.85	19.96	24.60
COVID-19		*H*_1_:*r* = 1	5.42	7.52	9.24	12.97
	Spot (Calcium carbide)	*H*_0_:*r* = 0	58.22	17.85	19.96	24.60
		*H*_1_:*r* = 1	4.81	7.52	9.24	12.97
	Spot (Calcium carbide	*H*_0_:*r* = 0	40.61	17.85	19.96	24.60
	of East China)	*H*_1_:*r* = 1	4.46	7.52	9.24	12.97

During	Spot (Ethylene)	*H*_0_:*r* = 0	26.07	17.85	19.96	24.60
COVID-19		*H*_1_:*r* = 1	2.87	7.52	9.24	12.97
	Spot (Calcium carbide)	*H*_0_:*r* = 0	32.36	17.85	19.96	24.60
		*H*_1_:*r* = 1	3.05	7.52	9.24	12.97
	Spot (Calcium carbide	*H*_0_:*r* = 0	44.09	17.85	19.96	24.60
	of East China)	*H*_1_:*r* = 1	2.72	7.52	9.24	12.97

After	Spot (Ethylene)	*H*_0_:*r* = 0	24.19	17.85	19.96	24.60
COVID-19		*H*_1_:*r* = 1	0.90	7.52	9.24	12.97
	Spot (Calcium carbide)	*H*_0_:*r* = 0	24.57	17.85	19.96	24.60
		*H*_1_:*r* = 1	1.18	7.52	9.24	12.97
	Spot (Calcium carbide	*H*_0_:*r* = 0	21.42	17.85	19.96	24.60
	of East China)	*H*_1_:*r* = 1	0.76	7.52	9.24	12.97

Note: The table presents the results of the Johansen cointegration test for PVC markets. The test is conducted by the trace test, and the critical values at different significance levels (10%, 5%, 1%) are provided for comparison. The null hypothesis $H_{0}:r=0$ represents that there is no cointegration relationship, while $H_{1}:r=1$ indicates the presence of one cointegration relationship.

## 4 Empirical analysis

### 4.1 Price discovery of PVC market

To assess short-term shocks in PVC futures and spot markets, we computed the error correction coefficients of the vector error correction model (VECM), and the results are presented in Table 6. The error correction term for PVC markets is defined as $ECT_{t}=x_{1,t}-x_{2,t}$, where $x_{1,t}$ and $x_{2,t}$ denote the logarithms of the future price and spot price, respectively. Our empirical findings reveal a bidirectional cointegration relationship between futures and spot markets before the conclusion of the pandemic. This implies that both PVC futures and spot prices can adjust to a non-equilibrium state. Notably, the coefficients in PVC spot markets are larger than those in futures markets, indicating a swifter adjustment speed to short-term shocks in spot markets. For instance, during the COVID-19 pandemic, the error correction coefficient for the PVC futures market is 0.0130, while the coefficient for the ethylene spot market is 0.0383. This suggests a positive adjustment effect of the error correction term on both futures and spot markets, with a more significant impact on PVC spot prices. However, post the COVID-19 pandemic, there is a unidirectional cointegration from calcium carbide spot markets to futures markets. Holding other variables constant, a 1% deviation of spot prices above their equilibrium relationship relative to futures prices is expected to lead to an average increase of 0.1479% and 0.1909% in subsequent period spot prices, respectively. The impact of the disequilibrium error on PVC spot prices becomes even more pronounced under these conditions.

**Table 6:** Error correction coefficients of VECM.

Market	Variable	All data	Before	During	After
			COVID-19	COVID-19	COVID-19
Futures market &	Futures	-0.0046***	-0.0062***	0.0130**	-0.1524*
Spot market		(0.0047)	(0.0046)	(0.0171)	(0.0785)
(Ethylene)	Spot	0.0101***	0.0064***	0.0383***	0.0977**
		(0.0020)	(0.0014)	(0.0093)	(0.0425)

Futures market &	Futures	0.0012***	-0.0042***	0.0366**	-0.1890
Spot market		(0.0063)	(0.0065)	(0.0202)	(0.1078)
(Calcium carbide)	Spot	0.0199***	0.0113***	0.0546**	0.1479*
		(0.0032)	(0.0022)	(0.0132)	(0.0610)

Futures market &	Futures	-0.0009***	-0.0095***	0.0355**	-0.1608
Spot market		(0.0061)	(0.0061)	(0.0203)	(0.1123)
(Calcium carbide	Spot	0.0179***	0.0106***	0.0636**	0.1909*
of East China)		(0.0036)	(0.0028)	(0.0145)	(0.0752)

Note: The table displays the error correction coefficients of the VECM for different time divisions in the PVC markets. The coefficients indicate the short-term adjustment to deviations from the long-term equilibrium. The values are reported as test statistics followed by p-values in parentheses. *** means p<0.01, ** means p<0.05, and * means p<0.1. If the test results are not significant, it indicates that there is no one-way cointegration relationship between the markets.

This study employs the CS model, IS model, improved IS model, and ILS model to calculate the price discovery relationship between PVC futures and spot markets since the inception of PVC futures trading. In the calculation of the information shares between PVC futures and spot markets, due to the Cholesky factorization method increasing the information share of the first market, this paper, when using the IS model, swaps the order of market price sequences in the VECM. The average value of market information shares is then used as the final information shares for both futures and spot markets. To ascertain the optimal lag order for cointegration analysis, we conducted a simultaneous assessment using multiple criteria, including the Akaike Information Criterion (AIC), Hannan-Quinn Information Criterion (HQ), Schwarz Information Criterion (SC), and Final Prediction Error (FPE). The selection of the optimal lag order for our model is determined based on the results derived from the Schwarz Information Criterion (SC). The outcomes of the lag order selection are presented in Table 7. The results of the price discovery relationship between PVC futures and spot markets are presented in Table 8.

**Table 7:** Lag order selection results.

Time division	Variable	AIC	HQ	SC	FPE
All data	Spot (Ethylene)	20	7	6	20
	Spot (Calcium carbide)	21	6	6	21
	Spot (Calcium carbide	16	6	6	16
	of East China)				

Before	Spot (Ethylene)	11	11	6	11
COVID-19	Spot (Calcium carbide)	11	7	7	11
	Spot (Calcium carbide	11	6	4	11
	of East China)				

During	Spot (Ethylene)	6	6	4	6
COVID-19	Spot (Calcium carbide)	6	3	3	6
	Spot (Calcium carbide	6	4	2	6
	of East China)				

After	Spot (Ethylene)	2	1	1	2
COVID-19	Spot (Calcium carbide)	2	2	1	2
	Spot (Calcium carbide	2	2	1	2
	of East China)				

Note: The table presents the lag order selection results for the VECM. The lag order is determined based on the criterion of AIC, HQ, FPE and SC. The optimal lag order is finally selected based on the outcomes of SC.

**Table 8:** Price discovery results of PVC markets.

Market	Model	Variable	All data	Before	During	After
				COVID-19	COVID-19	COVID-19
Futures market &	CS model	Futures	68.76%	50.86%	74.67%	39.05%
Spot market		Spot	31.24%	49.14%	25.33%	60.95%
(Ethylene)	IS model	Futures	87.64%	87.23%	94.38%	54.79%
		Spot	12.36%	12.77%	5.62%	45.21%
	ILS model	Futures	91.22%	97.76%	97.01%	78.15%
		Spot	8.78%	2.24%	2.99%	21.85%
	Modified	Futures	64.50%	50.11%	96.91%	36.57%
	IS model	Spot	35.50%	49.89%	3.09%	63.43%

Futures market &	CS model	Futures	87.52%	54.44%	59.86%	37.07%
Spot market		Spot	12.48%	45.56%	40.14%	62.93%
(Calcium carbide)	IS model	Futures	87.05%	84.38%	87.65%	50.89%
		Spot	12.95%	15.62%	12.35%	49.11%
	ILS model	Futures	47.90%	95.34%	95.77%	75.58%
		Spot	52.10%	4.66%	4.23%	24.42%
	Modified	Futures	79.05%	51.95%	83.69%	35.60%
	IS model	Spot	20.95%	48.05%	16.31%	64.40%

Futures market &	CS model	Futures	76.49%	45.99%	65.03%	46.40%
Spot market		Spot	23.51%	54.01%	34.97%	53.60%
(Calcium carbide	IS model	Futures	81.24%	71.05%	87.41%	55.03%
of East China)		Spot	18.76%	28.95%	12.59%	44.97%
	ILS model	Futures	63.92%	89.25%	93.30%	66.65%
		Spot	36.08%	10.75%	6.70%	33.35%
	Modified	Futures	69.76%	45.07%	95.22%	43.12%
	IS model	Spot	30.24%	54.93%	4.78%	56.88%

Note: The table presents the results of price discovery analysis in PVC markets using different models for various time divisions. The percentages represent the contribution of futures and spot markets to the price discovery process.

In the study of price discovery using the full set of PVC futures and spot price data, it was observed that the PVC futures market plays a dominant role in the price discovery process. According to the calculations of the CS model, the price discovery contributions of the PVC futures market in the ethylene-based spot market, the nationwide calcium carbide-based spot market, and the East China calcium carbide-based spot market were 68.76%, 87.52%, and 76.49% respectively. In contrast, the contribution of the spot market to price discovery was 31.24%, 12.48%, and 23.51% respectively. Overall, the PVC futures market's share in price discovery was significantly higher than that of the spot market, indicating that the PVC futures market's prices respond more sensitively to market information, adjusting to market information ahead of the spot market. In the analysis of the pre-COVID-19 pandemic period, the PVC futures prices dominated the price discovery process in both the ethylene-based and calcium carbide-based spot markets. In the CS model, the contribution of the PVC futures market to price discovery was 50.86% and 54.44% respectively. In the modified IS model, the contributions were 50.11% and 51.95% respectively. While the results of the CS model and the modified IS model were similar, the proportion of price discovery by the PVC futures market in this period was much lower than in the full data analysis. This suggests that before the pandemic, the price discovery capabilities of the PVC futures and spot markets were similar, with the futures market slightly ahead in terms of price discovery.

For the East China spot market, in the context of price discovery analysis, the PVC spot market in the East China region dominated the price discovery process before the pandemic. In both the CS and IS models, the contribution of the East China calcium carbide-based spot market to price discovery was 54.01% and 28.95% respectively. This resulted in a paradoxical situation in the model results. To address this, the modified IS model was employed to eliminate the correlation between the residual sequences of the two markets. The results of the modified IS model showed that the contribution of the East China calcium carbide-based spot market to price discovery was 54.93%, consistent with the CS model's calculation. The modified IS model effectively resolved the contradictory conclusions between the CS model and the original IS model.

From December 11, 2019, to December 4, 2022, which corresponds to the period of the COVID-19 pandemic in China, the PVC futures market took the lead in the price discovery process across the three spot markets. In the CS model, the contribution of the PVC futures market to price discovery was 74.67%, 59.86%, and 65.03% respectively. During the pandemic period's long-term equilibrium, the PVC futures market had the highest price discovery contribution in the ethylene-based spot market. According to the results of the modified IS model, the price discovery proportions for the PVC futures market were 96.91%, 83.69%, and 95.22% respectively. The similar results across the three spot markets during the pandemic indicate that, in the short-term equilibrium process, the PVC futures market had a greater contribution to price discovery compared to the spot markets.

On December 5, 2022, China declared the end of the COVID-19 pandemic. After the pandemic, the PVC spot market took the lead in the price discovery process. The nationwide ethylene-based spot market, calcium carbide-based spot market, and East China calcium carbide-based spot market's contributions to price discovery were 60.95%, 62.93%, and 53.60% respectively in the CS model. In the IS model, the price discovery proportions were 45.21%, 49.11%, and 44.97% respectively, which is opposite to the CS model's results. However, after removing the correlation between market residual sequences, the results of the modified IS model were 63.43%, 64.40%, and 56.88%, respectively. The modified IS model indicates that the PVC spot market had a greater contribution to price discovery after the pandemic, highlighting its dominant role in the price discovery process.

The economic significance of price discovery between futures and spot markets refers to the mutual influence and information transmission of prices for a commodity across different markets. When PVC futures are traded in both futures and spot markets, price discrepancies arise. These discrepancies reflect differences in the understanding of market supply and demand conditions, market expectations, and new information among participants in the futures and spot markets. The price spread between PVC markets encourages market participants to engage in arbitrage transactions, thereby driving futures and spot prices towards equilibrium. Before the end of the COVID-19 pandemic, the dominance of the PVC futures market in the price discovery process indicates that the PVC futures market operates efficiently, stabilizes market prices, and provides effective price references for the spot market. However, after the end of the pandemic, both PVC futures and spot markets experienced significant price volatility, with their price coefficients of variation being much higher than before and during the pandemic. As of June 9, 2023, the downstream PVC inventory in East China and South China warehouses stood at 394,000 tons, reaching historically high levels. This suggests a substantial inventory buildup in downstream markets where PVC is processed and sold. This could be indicative of strong downstream demand, as distributors and processing companies actively stock up to meet market demands. The new information is first responded to in the spot market rather than the futures market.

### 4.2 Robustness analysis

In order to further test the robustness of the price discovery results, we use the settlement prices of active PVC futures contracts and the closing prices of the Wind PVC Futures Price Index as the prices for the PVC futures market. These prices are then analyzed for price discovery in comparison with the market prices of China's PVC ethylene-based spot market, calcium carbide-based spot market, and East China calcium carbide-based spot market. The Wind PVC Futures Price Index is a price index calculated by weighting different PVC futures contracts based on their open interest. The results of the robustness analysis for PVC market price discovery are presented in Table 9 and Table 10, respectively.

**Table 9:** Robust analysis of PVC markets (Active futures contract settlement price).

Market	Model	Variable	All data	Before	During	After
				COVID-19	COVID-19	COVID-19
Futures market &	CS model	Futures	72.91%	53.31%	74.28%	31.67%
Spot market		Spot	27.09%	46.69%	25.72%	68.33%
(Ethylene)	IS model	Futures	84.59%	84.82%	90.74%	42.19%
		Spot	15.41%	15.18%	9.26%	57.81%
	ILS model	Futures	80.61%	95.99%	92.01%	71.26%
		Spot	19.39%	4.01%	7.99%	28.74%
	Modified	Futures	68.89%	51.54%	97.40%	25.12%
	IS model	Spot	31.11%	48.46%	2.60%	74.88%

Futures market &	CS model	Futures	85.80%	55.40%	66.48%	10.25%
Spot market		Spot	14.20%	44.60%	33.52%	89.75%
(Calcium carbide)	IS model	Futures	81.72%	79.38%	82.89%	31.76%
		Spot	18.28%	20.62%	17.11%	68.24%
	ILS model	Futures	35.35%	90.57%	85.65%	94.32%
		Spot	64.65%	9.43%	14.35%	5.68%
	Modified	Futures	75.51%	52.48%	93.72%	4.42%
	IS model	Spot	24.49%	47.52%	6.28%	95.58%

Futures market &	CS model	Futures	73.52%	47.31%	68.63%	20.96%
Spot market		Spot	26.48%	52.69%	31.37%	79.04%
(Calcium carbide	IS model	Futures	73.88%	65.36%	82.50%	37.64%
of East China)		Spot	26.12%	34.64%	17.50%	62.36%
	ILS model	Futures	50.91%	81.55%	82.28%	83.83%
		Spot	49.09%	18.45%	17.72%	16.17%
	Modified	Futures	66.20%	44.98%	89.57%	12.25%
	IS model	Spot	33.80%	55.02%	10.43%	87.75%

Note: The table presents the robustness analysis of PVC markets by using the active futures contract settlement prices. The percentages represent the contribution of futures and spot markets to the price discovery process.

**Table 10:** Robust analysis of PVC markets (PVC futures price index closing price).

Market	Model	Variable	All data	Before	During	After
				COVID-19	COVID-19	COVID-19
Futures market &	CS model	Futures	80.65%	60.95%	73.46%	44.13%
Spot market		Spot	19.35%	39.05%	26.54%	55.87%
(Ethylene)	IS model	Futures	90.96%	91.26%	94.27%	60.70%
		Spot	9.04%	8.74%	5.73%	39.30%
	ILS model	Futures	85.37%	97.81%	97.25%	79.27%
		Spot	14.63%	2.19%	2.75%	20.73%
	Modified	Futures	76.75%	56.30%	96.48%	42.15%
	IS model	Spot	23.25%	43.70%	3.52%	57.85%

Futures market &	CS model	Futures	94.44%	73.04%	59.83%	43.90%
Spot market		Spot	5.56%	26.96%	40.17%	56.10%
(Calcium carbide)	IS model	Futures	90.17%	91.00%	87.27%	56.94%
		Spot	9.83%	9.00%	12.73%	43.06%
	ILS model	Futures	22.56%	93.29%	95.49%	74.07%
		Spot	77.44%	6.71%	4.51%	25.93%
	Modified	Futures	93.08%	65.65%	85.07%	42.38%
	IS model	Spot	6.92%	34.35%	14.93%	57.62%

Futures market &	CS model	Futures	95.21%	52.77%	64.18%	54.28%
Spot market		Spot	4.79%	47.23%	35.82%	45.72%
(Calcium carbide	IS model	Futures	86.43%	75.46%	87.14%	60.98%
of East China)		Spot	13.57%	24.54%	12.86%	39.02%
	ILS model	Futures	9.32%	88.33%	93.47%	63.41%
		Spot	90.68%	11.67%	6.53%	36.59%
	Modified	Futures	84.20%	50.74%	94.82%	50.36%
	IS model	Spot	15.80%	49.26%	5.18%	49.64%

Note: The table presents the robustness analysis of PVC markets using the closing prices of PVC futures price index. The percentages represent the contribution of futures and spot markets to the price discovery process.

When using active PVC futures contracts as the prices for the PVC futures market in empirical analysis, the price discovery results are generally consistent with the results obtained using the closing prices of active PVC futures contracts. Notably, after the end of the COVID-19 pandemic, the price discovery conclusions of the IS model are consistent with the CS model, indicating that the spot market played a dominant role in the price discovery process, and there were no contradictory outcomes between the IS and CS models. When using the closing prices of the Wind PVC Futures Price Index as the prices for the PVC futures market, the price discovery results show slight differences compared to the calculations in Section 3.2. Before the outbreak of the COVID-19 pandemic, the contribution of the East China calcium carbide-based spot market prices to price discovery was 47.23% in the CS model and 49.26% in the modified IS model. This suggests that PVC futures prices dominated the price discovery process before the pandemic. Meanwhile, after the end of the pandemic, the contribution of the East China calcium carbide-based spot market prices to price discovery was 45.72% in the CS model and 49.64% in the modified IS model. This indicates that after the pandemic, the dominant market in the price discovery process in the East China region was the PVC futures market. According to the results of the modified IS model, the contributions of price discovery for PVC futures and spot markets were very close, with percentages of 50.36% and 49.64%, respectively.

### 4.3 Impulse responses

**Figure 4:**
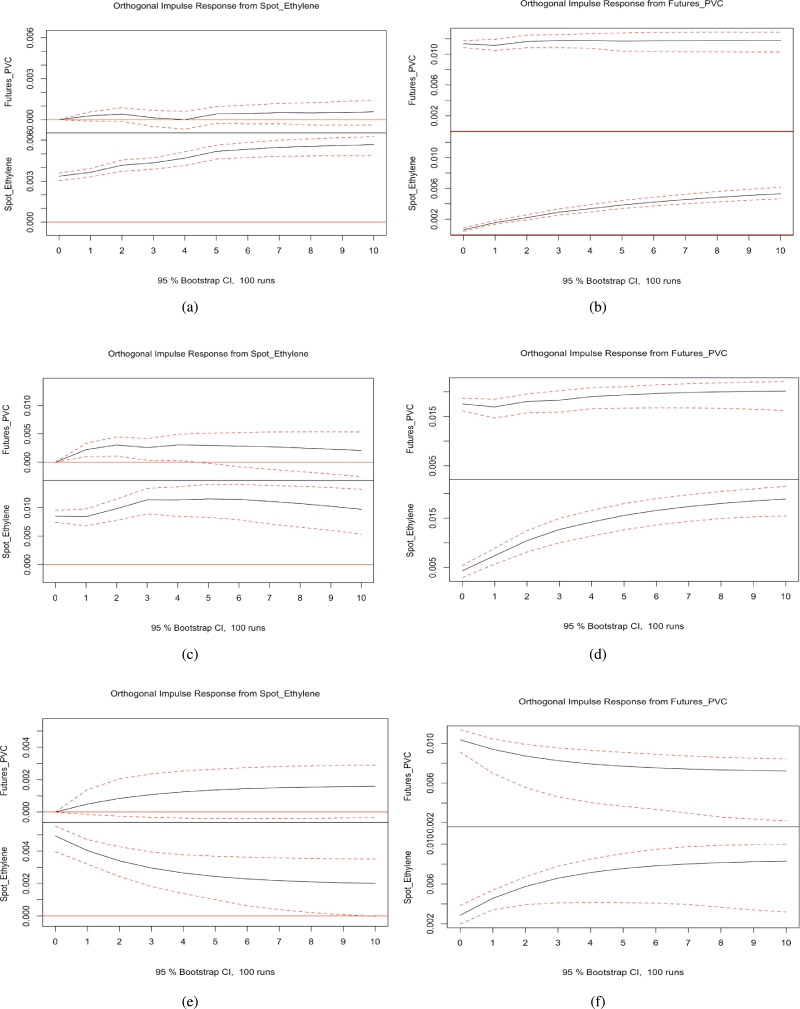
(a) (b): The impulse response functions of PVC futures and the ethylene-based spot market before the COVID-19 pandemic. (c) (d) The impulse response functions of PVC futures and the ethylene-based spot market during the COVID-19 pandemic. (e) (f): The impulse response functions of PVC futures and the ethylene-based spot market after the COVID-19 pandemic.

**Figure 5:**
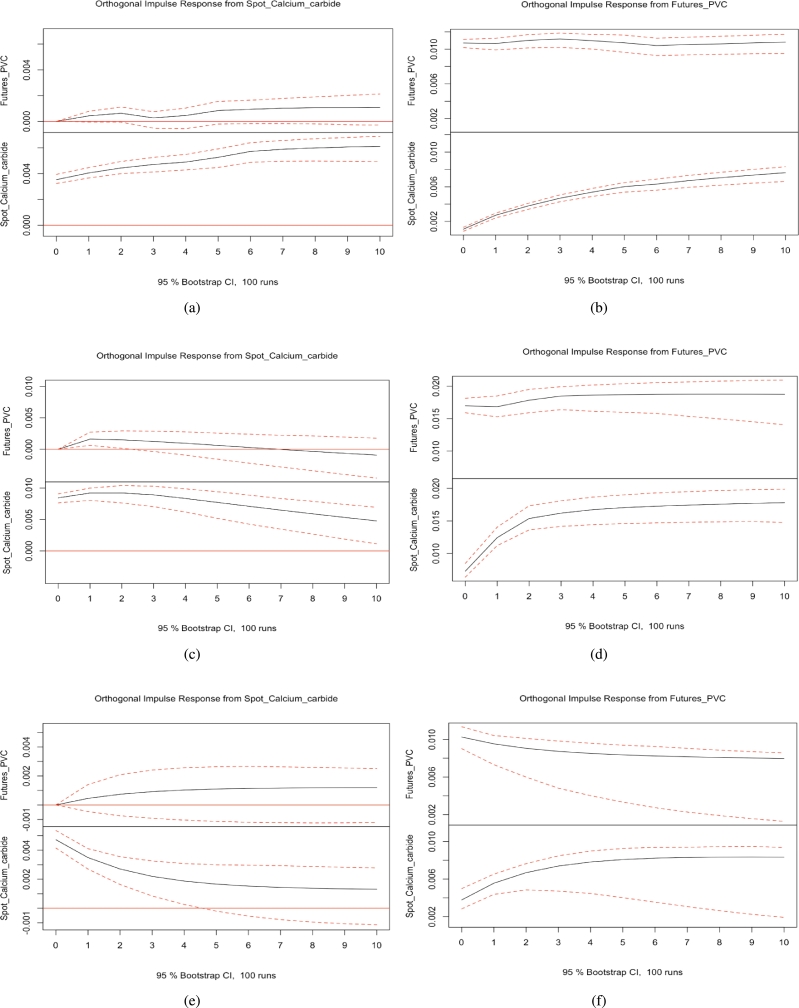
(a) (b): The impulse response functions of PVC futures and the calcium carbide spot market before the COVID-19 pandemic (c) (d) The impulse response functions of PVC futures and the calcium carbide spot market during the COVID-19 pandemic. (e) (f): The impulse response functions of PVC futures and the calcium carbide spot market after the COVID-19 pandemic.

**Figure 6:**
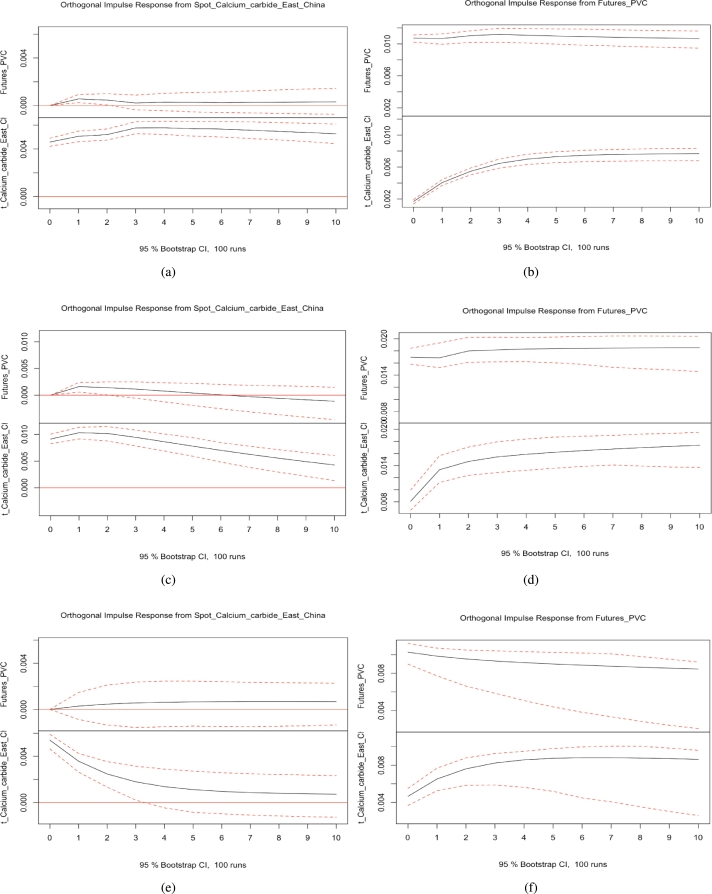
(a) (b): The impulse response functions of PVC futures and the calcium carbide spot market of East China before the COVID-19 pandemic. (c) (d) The impulse response functions of PVC futures and the calcium carbide spot market of East China during the COVID-19 pandemic. (e) (f): The impulse response functions of PVC futures and the calcium carbide spot market of East China after the COVID-19 pandemic.

Fig. 4 (a)-(f) demonstrate the impulse response functions of PVC futures and the ethylene-based spot market. Fig. 5 (a)-(f) demonstrate the impulse response functions of PVC futures and the calcium carbide spot market. Fig. 6 (a)-(f) demonstrate the impulse response functions of PVC futures and the calcium carbide spot market of East China before the COVID-19 pandemic. The corresponding details are given in the captions of the figures. Through the calculation of impulse response functions for PVC futures and spot markets, we find no clear evidence of asymmetrical effects between PVC futures and spot prices. Symmetrical effects in impulse response refer to a scenario in time series analysis where a system's response to positive and negative impulses exhibits similar or mirrored characteristics. If the system's response to positive and negative shocks is symmetrical, then the shapes of the impulse response function in both positive and negative directions should be similar. This indicates that the system's dynamic characteristics or response patterns are similar when facing positive and negative shocks. Conversely, asymmetrical effects in impulse response signify a phenomenon in time series analysis where the impact of positive and negative fluctuations from the same shock differs on the system. The existence of asymmetrical effects may suggest varying sensitivity of the system to positive and negative impulses or the presence of different dynamic adjustment mechanisms in different shock directions.

We computed impulse response functions for PVC futures and spot markets, as well as spot markets using the ethylene method, the calcium carbide method, and the eastern region spot market using the calcium carbide method, before and after the pandemic. Our findings reveal that when a shock is applied to the PVC spot market, it generates a sustained positive impact on the PVC futures market, indicating that if PVC spot market prices rise, PVC futures market prices will also increase. Similarly, when a shock is applied to the PVC futures market, it has a sustained positive impact on the PVC spot market, demonstrating that if PVC futures market prices rise, PVC spot market prices will also rise. During the pandemic, when a shock is applied to the PVC spot market using the calcium carbide method, the positive impact on the PVC futures market strengthens in the first period but gradually weakens from the second period onward, producing a subtle negative impact. Overall, no apparent asymmetrical effects exist between PVC futures and spot prices.

## 5 Conclusion

This paper conducted an analysis of the price discovery capabilities between China's PVC futures and spot markets. The analysis began with a comprehensive study using the full set of PVC futures and spot data since the introduction of PVC futures products. Overall, the PVC futures market exhibited stronger price discovery capabilities compared to the PVC spot market, indicating its dominant role in the price discovery process. The futures market could quickly respond to new information entering the PVC market and reflect it in futures prices. Based on the timing of the outbreak and end of the COVID-19 pandemic in China, the study dynamically analyzed the price discovery process before, during, and after the pandemic. The results indicated that after the pandemic, the PVC spot market took the lead in the price discovery process. Before and during the pandemic, the price discovery process was mainly dominated by the PVC futures market. The complex relationship between the price guidance of China's PVC futures and spot markets before and after the pandemic further complicates the challenge of PVC product hedging. Based on our results, we also provide some suggestions from the perspectives of regulation and policy, which are presented in Table 11. In future research, considering multidimensional micro-level data such as enterprise inventory, commercial stock levels, upstream and downstream costs, import and export quantities and prices, frequency of operation stoppages, and time, would provide a more comprehensive understanding of the price discovery relationship.

**Table 11:** Suggestions for PVC Futures Market.

Policy and Regulatory	Suggestions
Regulatory Framework	Regularly review and adapt the regulatory framework governing the PVC futures market to ensure its alignment with the evolving dynamics of the industry.
Information Transparency	Implement measures to enhance information transparency in both PVC futures and spot markets.
Risk Management Strategies	Encourage market participants, especially PVC manufacturers and consumers, to adopt robust risk management strategies that account for the unique challenges posed by the calcium carbide production method.
Market Integration and Global Collaboration	Foster collaboration with international counterparts to address global disparities in PVC production methods and market prices.
Research and Data Accessibility	Promote and support research initiatives that delve deeper into the price discovery mechanisms of PVC futures and spot markets, especially during macroscopic events.
Adaptive Preparedness	Develop adaptive strategies and contingency plans for the PVC futures market, considering the lessons learned from the impact of the big macro event.
Market Collaboration Platforms	Establish platforms or forums that facilitate collaboration and information exchange among market participants, regulatory bodies, and research institutions.

Note: The table presents the suggestions in terms of policy and regulatory.

## Funding

The research is funded by the project from the Science and Technology Development Center of the Ministry of Education (No. 2021IT02006).

## Data availability statement

Data will be made available on request.

## CRediT authorship contribution statement

**Yunyan Wu:** Writing – review & editing, Writing – original draft, Visualization, Resources, Project administration, Methodology, Investigation, Formal analysis, Data curation, Conceptualization. **Jiaqi He:** Writing – review & editing, Writing – original draft, Visualization, Software, Resources, Formal analysis, Data curation, Conceptualization. **Linfei Xiong:** Writing – review & editing, Writing – original draft, Validation, Supervision, Methodology, Formal analysis.

## Declaration of Competing Interest

The authors declare that they have no known competing financial interests or personal relationships that could have appeared to influence the work reported in this paper.
